# Pineal region teratoma with metastases in uncommon locations: a case report

**DOI:** 10.1186/s13256-022-03454-z

**Published:** 2022-06-23

**Authors:** Bayan AlRefaei, Taj Al Haj Husain, Ricarda Alwaw, Fatema Alzahraa Salama, Ghassan Hamzeh

**Affiliations:** 1 Faculty of Medicine, Damascus University, Damascus, Syria; 2 Department of Neurology, Al Assad Damascus University Hospital, Damascus, Syria

**Keywords:** Case report, CNS tumors, Pineal region, Teratoma, Metastasis

## Abstract

**Background:**

We report a rare case in medical literature of a patient with pineal gland teratoma and uncommon metastases. Usually, metastases of this kind of tumor are located in several organs such as lung and breast, but here we found metastases to the spinal cord and vertebrae.

**Case presentation:**

A 35-year-old Asian white man presented with diplopia and acute neural symptoms in the lower limbs such as numbness, tingling, and paralysis. His medical history was notable for pineal teratoma, treated 1 year previously with surgery, radiotherapy, and chemotherapy. Physical examination of the lower limbs showed absent reflexes and sensation with muscle power scale score of 1 in both limbs. Magnetic resonance imaging of brain and spine revealed many lesions in various locations, most compatible with neural, spinal, and vertebral metastases. Unfortunately, the patient died suddenly before any intervention was carried out.

**Conclusion:**

It is extremely rare for pineal region teratoma to metastasize to the spinal cord and vertebrae, thus more vigilant observation and examination should be provided to patients with pineal teratoma to detect any new lesions and prevent them from becoming dangerous.

## Introduction

The pineal gland lies posteriorly in the midline between the surface of the third ventricle and the midbrain, covered by the pia mater. It is responsible for the synthesis and secretion of melatonin that regulates the sleep–wake cycle [1]. Germ cell tumors (GCTs) are intracranial neoplasms that include germinomas, teratomas, choriocarcinomas, yolk sac or endodermal sinus tumors, embryonal carcinomas, and mixed germ cell tumors [2].

Clinical signs can vary from increased intracranial pressure (ICP), visual disorders, endocrine abnormalities, and diabetes insipidus (DI), in addition to Parinaud’s syndrome [3].

To the best of the authors’ knowledge, only one study has reported pineal teratoma with vertebral metastasis [4]. We report herein a rare case of an adult patient with uncommon metastases after pineal teratoma treatment.

## Case presentation

A 35-year-old malnourished Asian white man was referred to our hospital with a main complaint of acute paralysis, accompanied by diplopia, tingling and numbness in the lower limbs, and urinary retention for the last 5 days.

The patient had previously been diagnosed with pineal teratoma 1 year previously; craniotomy was done to excise the tumor, and he underwent radiotherapy and chemotherapy, with the last dose 3 months previously. He has unremarkable family history for diseases or cancers.

His physical examination showed absent reflexes and sensation in both lower limbs with MRC muscle power scale score of 1 in both lower limbs. He was slightly pale and fatigued due to malnutrition, but his vital signs were normal.

**Fig. 1:**
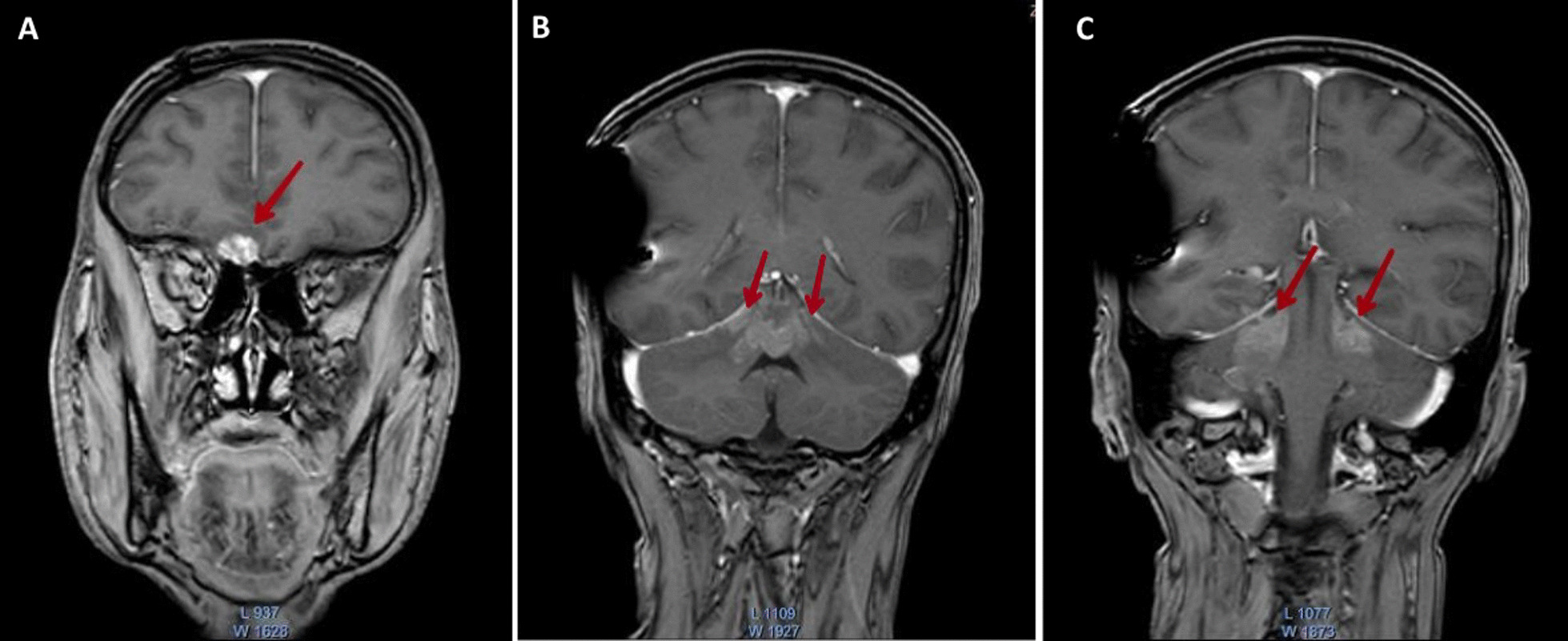
T1-weighted postcontrast MRI images show brain metastases: **a** one enhancing lesion in the right temporal lobe in transversal view, **b** lesions around pons Varolii in coronal view, and **c** lesions around the midbrain in coronal view

**Fig. 2:**
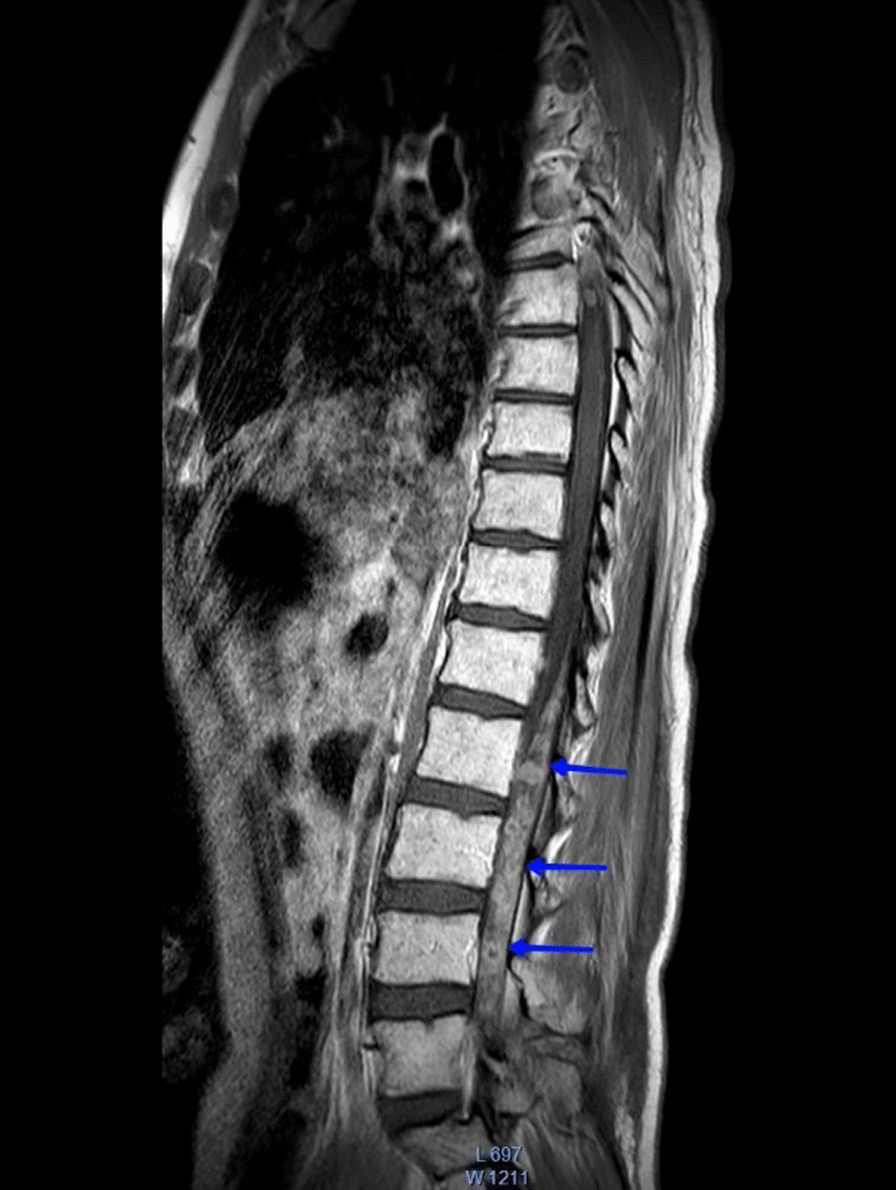
T1-weighted postcontrast MRI in sagittal view showing metastases called drop metastasis (blue arrows) to the medullary cone region

**Fig. 3:**
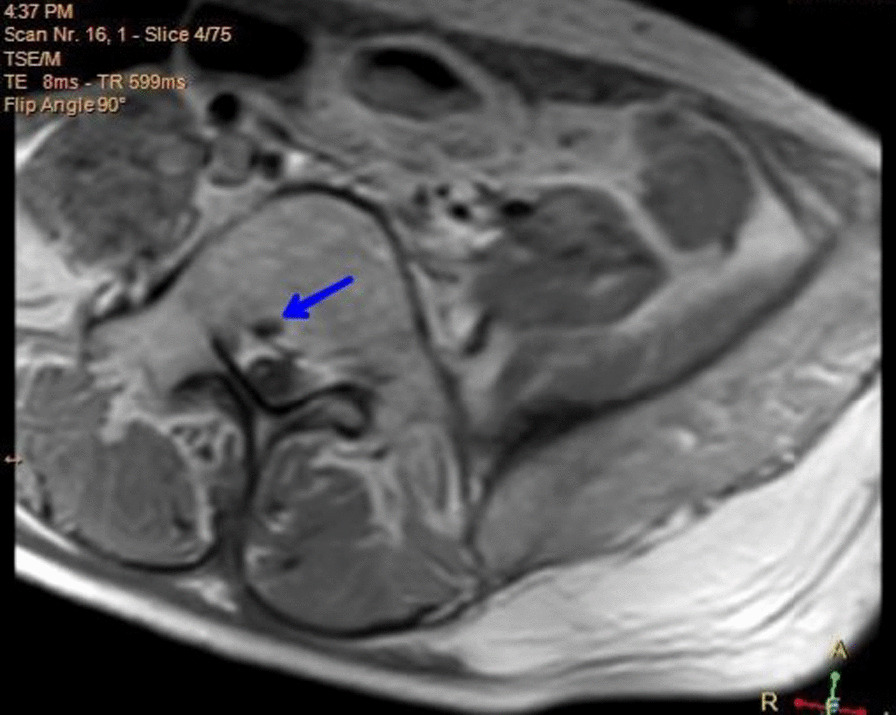
T1-weighted postcontrast MRI transversal view showing metastasis (blue arrow) to the second lumber vertebra

Complete blood count (CBC) showed low hemoglobin level of 10.8 g/dL but high levels of lactate dehydrogenase (LDH) and creatinine phosphokinase (CPK) at 830 U/L and 1864 U/L respectively. He was scheduled for brain and spine MRI, which revealed one lesion in the right temporal lobe (Fig. 1a), many lesions around pons Varolii (Fig. 1b), many lesions around the midbrain (Fig. 1c), many lesions in the medullary cone region in the spine (Fig. 2), and finally, one lesion in the second lumbar vertebra (Fig. 3). The first diagnosis was recurrent pineal region teratoma with neural and extraneural metastases. Unfortunately the patient died unexpectedly and quickly due to his serious medical condition before any intervention was carried out.

## Discussion

The pineal gland is a neuroendocrine gland responsible for melatonin production and release into the bloodstream [11]. Tumors in the pineal region are fairly rare, accounting for less than 1% of primary intracranial neoplasms of the central nervous system (CNS). They are often diagnosed at early age, between 10 and 21 years in 68% of cases. Also, they affect males more than females [2, 5].

Intracranial germ cell tumors (GCTs) can be divided into germinomas and nongerminomatous germ cell tumors (NGGCTs), which include five major groups of teratomas (mature, immature, and teratoma with malignant differentiation), choriocarcinomas, yolk sac or endodermal sinus tumors, embryonal carcinomas, and mixed GCTs (intermediate- and poor-prognosis groups) [2–10].

A series of 370 patients aged 3–73 years with pineal tumors found that the most common type was germinoma (27%), followed by astrocytoma (26%) then pineoblastoma (12%), pineocytoma (12%), ependymoma (4.3%), teratoma (4.3%,), metastasis, ganglioglioneuroma, lymphoma, meningioma, and pineal cyst (2.7%), mixed embryonal cell tumor (embryonal carcinoma) (1.6%), choriocarcinoma (1.1%), and oligodendroglioma (0.54%) [12].

In our case, the patient had pineal teratoma with age of 34 years at time of diagnosis, and recurrent teratoma with spinal and vertebrae metastasis when he came to our hospital. Just one report of pineal region metastasis to spinal cord was found in literature.

The most frequent location of GCTs is the pineal and neurohypophyseal (suprasellar) region, while they occur less commonly in basal ganglia or other brain localities [6].

Clinical signs depend on the tumor location and size and patient age. Pineal region tumors usually cause obstructive hydrocephalus leading to increased intracranial pressure (ICP), revealed by headache, nausea, vomitus, and somnolence.

Other possible symptoms of a tumor in the pineal region are visual disorders, endocrine abnormalities, sexual dysfunction, growth failure, puberty delay, ataxia, seizures, and behavioral changes.

Many patients develop diabetes insipidus (DI) because of dysfunction of neurohypophysis.

Parinaud’s syndrome is present in half of such patients, causing paralysis of upward gaze, while paralysis of downward gaze is less frequent, similarly to convergence disorder and/or convergence–retraction nystagmus [1, 3].

Computed tomography (CT) and magnetic resonance imaging (MRI) are the radiological examination modalities of choice in the diagnostic strategy, in addition to the presence of specific markers produced by GCTs such as alpha-fetoprotein (AFP), beta-human chorionic gonadotropin (beta-hCG), and placental alkaline phosphatase (PLAP). However, the final diagnosis depends on histological examination by biopsy, and it is important to choose the correct management [6–8].

Chemotherapy and radiation efficiently treat germinomas. On the other hand, surgical resection alone can treat teratomas. Meanwhile, mixed GCTs may require a combination of all the treatment choices mentioned before [10].

Germinomas and mature teratomas have the best recovery and life expectancy [9].

Based on the discussion above and due to the sudden death of the patient, the main diagnosis is recurrent pineal teratoma with spinal and vertebrae metastases. Because the tumor is at an advanced stage and neurological symptoms are caused by metastases, such patients with teratoma should be observed with frequent examination of both brain and spinal cord to detect any potential metastases.

## Conclusion

We report a rare case of recurrent pineal region teratoma metastases to the spinal cord and vertebrae. We believe that more vigilant observation and examination should be provided to patients with pineal teratoma to detect any new lesions and prevent them from becoming dangerous.

## Data Availability

Not applicable.

## Abbreviations

GCTs: Germ cell tumors
ICP: Increased intracranial pressure
DI: Diabetes insipidus
NGGCTs: Nongerminomatous germ cell tumors
CT: Computed tomography
MRI: Magnetic resonance imaging
AFP: Alpha-fetoprotein
Beta-hCG: Beta-human chorionic gonadotropin
PLAP: Placental alkaline phosphatase
